# Structural MRI phenotyping in Alzheimer’s disease: Comparison of visual rating scales, volumetry, and cortical thickness in a Serbian single-centre cohort

**DOI:** 10.17305/bb.2026.13974

**Published:** 2026-04-07

**Authors:** Aleksandra Aracki-Trenkic, Milica Živanović, Vuk Milošević, Jelena Bašić, Dunja Radovanović, Marina Malobabić

**Affiliations:** 1 Department of Radiology, Clinical Center Niš, Niš, Serbia; 2 Faculty of Medicine, University of Niš, Niš, Serbia; 3 Clinic of Neurology, Clinical Center Niš, Niš, Serbia; 4 Department of Biochemistry, Faculty of Medicine, University of Niš, Niš, Serbia

**Keywords:** Alzheimer’s disease, magnetic resonance imaging, brain volumetry, visual rating scales.

## Abstract

Alzheimer’s disease (AD) is characterized by a heterogeneous clinical course, and magnetic resonance imaging (MRI)-based phenotyping has increasingly been utilized to elucidate this variability. The literature recognizes four predominant MRI phenotypes: typical, hippocampal-sparing, limbic-predominant, and minimal-atrophy. However, the compatibility of various MRI phenotyping methods remains insufficiently defined. This study aimed to assess the concordance between MRI phenotyping methods within a Serbian cohort consisting of 40 subjects. Four MRI phenotyping approaches were employed: scale-based, adjusted scale-based, volume-based, and thickness-based. The scale-based method exhibited moderate agreement with the adjusted scale-based approach and high concordance with volumetric methods. In contrast, the relationship between scale- and thickness-based phenotyping was less clear. The lack of significant agreement with demographic variables, along with the observed differences across clinical dementia rating (CDR) domains, further underscored the clinical heterogeneity among phenotypes. Overall, these findings suggest that visual scale-based MRI phenotyping may serve as a practical approach in resource-limited clinical settings where advanced methods are unavailable. However, the results must be interpreted with caution and require validation in larger independent cohorts. Further research is necessary to clarify the relationship between scale- and thickness-based phenotyping across different disease stages and to investigate discrepancies in demographic, apolipoprotein E (APOE)-related, and clinical phenotype patterns in this Serbian sample compared to other populations.

## Introduction

Neurodegenerative causes of dementia are characterized by distinct patterns of atrophy detectable through magnetic resonance imaging (MRI). The classic MRI atrophy pattern in Alzheimer’s disease (AD) predominantly involves the medial temporal lobe and parietal region [1, 2]. Due to the heterogeneity in the clinical presentation of AD, several major clinical phenotypes have been identified. The most prevalent phenotype is marked by significant episodic memory impairment, while other phenotypes are defined by non-amnestic cognitive and behavioral deficits. These non-amnestic forms include dysexecutive AD, the behavioral variant of AD, posterior cortical atrophy, and the logopenic variant of primary progressive aphasia [3]. Previous studies have demonstrated that specific atrophy patterns can be recognized across these clinical phenotypes [4]. Furthermore, neuropathological research has identified distinct patterns of regional atrophy associated with the accumulation of neurofibrillary tangles (NFTs), which are graded using the Braak grading system, aligning with Braak stages III and IV [5]. These neuropathological patterns provide a biological basis for the specific atrophy patterns observed *in vivo* via MRI. A neuropathological study by Murray et al. [6] distinguished three subtypes of AD: typical, hippocampal sparing, and limbic predominant. Subsequent MRI studies identified four patterns: typical, hippocampal sparing, limbic predominant, and minimal atrophy [7–9]. Differentiating these four phenotypes enables the *in vivo* recognition of specific forms of AD, characterized by distinct clinical features, progression rates, and demographic characteristics [7, 9]. Various authors have conducted phenotyping using postmortem study results, volumetry, cortical thickness, voxel-based and surface-based morphometry, and visual and automated visual rating scales of atrophy [9–11]. Some researchers, such as Ferreira et al. [9], compared MRI phenotypes derived from visual rating scales with those based on cortical thickness. Li et al. [12] analyzed surface and voxel-based morphometric parameters. However, despite the variety of MRI-based approaches, no consensus exists on which phenotyping method yields the most clinically relevant results. Additionally, in low- and middle-income countries, many advanced MRI techniques are not routinely employed in clinical practice, highlighting the necessity of validating more accessible methods. We hypothesize that AD phenotyping based on straightforward visual MRI scales will demonstrate significant agreement with automated volumetric methods. Moreover, most studies to date have focused on Western European and North American populations, with limited data available on Southeast European populations, including Serbia. Therefore, we aim to compare MRI phenotyping approaches for AD and their association with dementia severity in the Serbian population.

## Materials and methods

### Participants

This study included 40 patients with a clinical diagnosis of dementia due to probable AD, confirmed by positive cerebrospinal fluid (CSF) biomarkers indicating underlying AD pathophysiology. The exploratory nature of this study, due to the small sample size and descriptive aims, necessitates that the results be regarded as hypothesis-generating.

The clinical diagnosis adhered to the 2011 National Institute on Aging–Alzheimer’s Association (NIA-AA) criteria for AD [13]. Participants were recruited from the Neurology Clinic of the University Clinical Centre Niš, Serbia. A comprehensive clinical assessment was conducted, including a detailed medical history obtained from both patients and caregivers. Standard laboratory investigations were performed to exclude secondary causes of cognitive impairment. Patients with significant neurological disorders other than AD, large territorial strokes, brain tumors, or severe psychiatric disorders were excluded from the study. Mild vascular changes consistent with age-related small vessel disease were not considered exclusion criteria.

Cognitive screening was conducted using the Mini-Mental State Examination (MMSE), followed by a detailed neuropsychological assessment. Disease severity was evaluated using the Clinical Dementia Rating (CDR) scale, with global CDR scores and CDR Sum of Boxes (CDR-SOB) calculated for each patient. Cognitive impairment severity was categorized according to MMSE scores as mild (MMSE ≥20), moderate (MMSE 10–19), and severe (MMSE <10).

### Genotyping and biomarker analyses

Lumbar puncture was performed on all participants, with CSF biomarkers analyzed using fully automated electrochemiluminescence immunoassays on the Elecsys platform (Roche Diagnostics, Mannheim, Germany), including Elecsys^®^ β-Amyloid (1–42) CSF, Elecsys^®^ Phospho-Tau (181P) CSF, and Elecsys^®^ Total-Tau CSF. All analyses were conducted following the manufacturer’s instructions using standardized laboratory procedures. CSF biomarker positivity for Alzheimer’s disease pathology was defined according to the NIA-AA research framework for the biological definition of AD [14]. Participants were classified as CSF-positive AD when their biomarker profile indicated amyloid and tau positivity (A+T+). Amyloid positivity (A+) was defined as CSF amyloid beta 42 (Aβ42) < 1030 pg/mL, while tau pathology (T+) was indicated by phosphorylated tau at threonine 181 (p-tau181) > 27 pg/mL. Additionally, the phosphorylated tau 181/Aβ42 (p-tau181/Aβ42) ratio (> 0.023) served as a supportive indicator of the AD biomarker profile.

Genomic DNA was isolated from whole blood collected in ethylenediaminetetraacetic acid (EDTA)-containing tubes. Apolipoprotein E (APOE) genotyping was performed using real-time polymerase chain reaction (PCR) to determine APOE ɛ4 allele carrier status, following a previously published protocol by our research group [15].

All patients were referred for a brain MRI as part of the diagnostic workup.

### Magnetic resonance imaging

The study was conducted at the Radiology Centre of the University Clinical Centre Niš, utilizing a 3T MRI (SIGNA PIONEER, General Electric (GE) Healthcare, USA). An established protocol for dementia at our institution [2] was employed, summarized in Table 1. For this study, we utilized two sequences from the protocol: 1) A 3D T1-weighted (T1w) sequence (voxel size 1×1×1 mm) for automated volumetric and cortical thickness analysis using the VolBrain pipeline, as well as for visual assessment of frontal subscore of the global cortical atrophy scale (F-GCA) and Koedam scale; 2) A high-resolution T1w (2 mm) paracoronal sequence parallel to the hippocampal long axis for assessing medial temporal atrophy. Four MRI phenotyping approaches were investigated and compared: scale-based, adjusted-scale-based, volume-based, and thickness-based methods.

**Table 1:** Standardized MRI protocol for dementia assessment at our institution

**Sequences**	**Orientation**	**Purpose/Evaluation**	**Slice thickness**	**Voxel size**
3D T1 Bravo	Axial	F-GCA and Koedam scales	1 mm	1 × 1 × 1 mm
T1W	Paracoronal – parallel to the hippocampal long axis	MTA scale	2 mm	0.4 × 0.4 × 2 mm
T2W FRFSE	Paracoronal – parallel to hippocampal long axis	Hippocampal signal changes	2 mm	0.4 × 0.4 × 2 mm
T2W PROP	Axial	Structural changes	2 mm	0.6 × 0.6 × 2 mm
3D FLAIR	Axial	Structural changes	2 mm	0.5 × 0.5 × 2 mm
3D SWAN	Axial	Vascular burden	3 mm	0.6 × 0.6 × 3 mm
DWI TENSOR	Axial	Restricted diffusion	4 mm	1 × 1 × 4.0 mm
3D ASL	Axial	Perfusion abnormalities	4 mm	2 × 2 × 4 mm

Abbreviations**:** 3D, three-dimensional; ASL, arterial spin labeling; BRAVO, brain volume imaging; DTI, diffusion tensor imaging; DWI, diffusion-weighted imaging; FLAIR, fluid-attenuated inversion recovery; FRFSE, fast recovery fast spin echo; PROP, periodically rotated overlapping parallel lines with enhanced reconstruction (PROPELLER); SWAN, susceptibility-weighted angiography; T1W, T1-weighted imaging; T2W, T2-weighted imaging.

### Visual scales assessment

To define the scale-based MRI phenotype, regional atrophy was evaluated using the medial temporal lobe atrophy scale (MTA) [16] for medial temporal regions, the F-GCA (Pasquire scale) [17] for frontal atrophy, and the Koedam scale [18] for posterior atrophy.

MTA scores ranged from 0 to 4, assessing collateral sulcus dilation, hippocampal size, and temporal horn dilation. The F-GCA score ranged from 0 to 3, reflecting bilateral frontal sulcus dilation and gyral atrophy. The Koedam scale, scored 0–3 in the sagittal plane, evaluates sulcal widening and gyral atrophy in posterior cortical regions. All scales were assessed for both the left and right sides. A region was classified as abnormal if its score met or exceeded the defined pathological threshold.

Two approaches were employed to define scale-based MRI phenotype. The first relied on commonly used cut-off values, selected for their simplicity and clinical applicability [2, 16–19]. In this approach, MTA was considered pathological at ≥2 unilaterally in individuals younger than 75 years or bilaterally in those aged 75 years or older. For F-GCA, pathology was defined as ≥2 for individuals under 75 years and ≥3 for those over 75 years. For Koedam, a pathological score was >1 if detected on at least one side, regardless of age.

**Figure 1.**
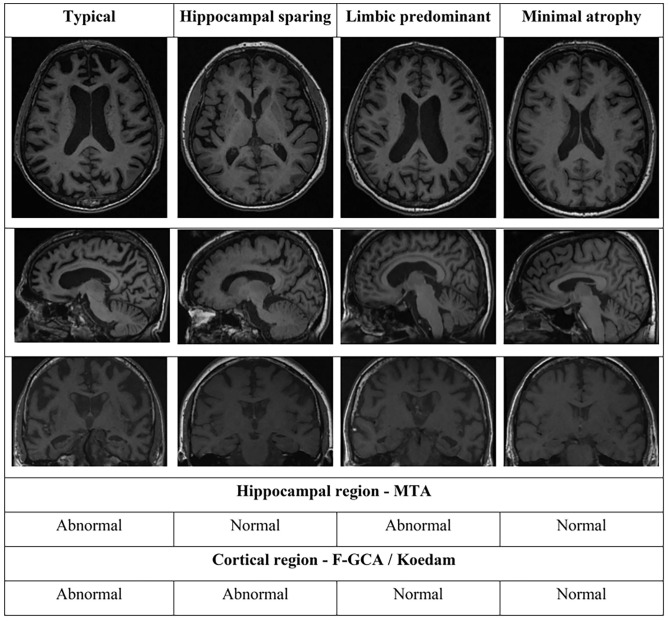
**Representative T1-weighted MRI examples of the four scale-based phenotypes identified in our cohort.** Columns (left to right) show the typical, hippocampal sparing, limbic predominant, and minimal atrophy phenotypes. For each representative case, axial 3D T1w images (top row), sagittal 3D T1w images (middle row), and paracoronal T1w images oriented parallel to the long axis of the hippocampus (bottom row) are shown. Phenotype classification was based on visual assessment of medial temporal atrophy (MTA), frontal cortical atrophy using the frontal subscore of the global cortical atrophy scale (F-GCA), and posterior atrophy (PA; Koedam scale). The typical phenotype was defined by pathological MTA together with pathological F-GCA and/or PA; hippocampal sparing by normal MTA with pathological F-GCA and/or PA; limbic predominant by pathological MTA with normal F-GCA and PA; and minimal atrophy by normal MTA, F-GCA, and PA. The same visual rating scales were used for both the standard scale-based and adjusted scale-based phenotyping approaches. All images were obtained from study participants and fully anonymised in accordance with the study ethical approval. Abbreviations: F-GCA, frontal subscore of global cortical atrophy; MRI, magnetic resonance imaging; MTA, medial temporal atrophy; PA, posterior atrophy; T1w, T1-weighted.

The second approach applied age (early-onset Alzheimer’s disease (EOAD) < 65 years) and APOE genotype-adjusted cut-offs, following the recommendations of Ferreira et al. [20] (Figure 1). The final score was calculated as the average of the left- and right-hemisphere ratings. In APOE ɛ4 allele carriers, the pathological threshold for MTA was defined as a score ≥1.5 for age groups 45–64 and 65–74 years, ≥2 for the 75–84 age group, and ≥2.5 for those older than 85 years. For the F-GCA and Koedam scales, the pathological finding was defined as a score ≥1 in all age groups. In EOAD patients who are not APOE ɛ4 allele carriers, the pathological threshold for MTA was ≥2 for ages 45–64 and 65–74 years, while in older age groups (75–84 and >85 years), the threshold was ≥3. For the F-GCA scale, a pathological finding is defined as a score ≥1 in younger age groups or ≥2 in subjects older than 75 years, while for the Koedam scale, a pathological finding is defined as a score ≥1, regardless of age.

In defining scale-based and adjusted scale-based phenotyping, we followed Ferreira et al. [20]. The typical phenotype includes pathological MTA, F-GCA, and/or posterior atrophy (PA). The hippocampal sparing phenotype features normal MTA with abnormal F-GCA and/or PA. The limbic predominant phenotype displays abnormal MTA with normal F-GCA and PA. The minimal atrophy phenotype presents with normal MTA, F-GCA, and PA (Figure 1).

Visual ratings were independently performed by an experienced neuroradiologist with over 15 years of experience in assessing patients with neurodegenerative diseases. The MTA scale was evaluated using high-resolution T1w paracoronal sequences oriented parallel to the hippocampus’s long axis. The F-GCA scale was assessed using the frontal subscore of 3D T1w sequences reviewed in all three planes. Posterior atrophy, according to the Koedam scale, was evaluated on sagittal reconstructions of the 3D T1w sequence. The rater was blinded to the results of automated volumetric and cortical thickness analyses obtained from the VolBrain platform. Given that visual rating scales are commonly used in routine practice, and the primary aim of this study was to compare MRI phenotyping approaches rather than to validate the scales themselves, inter-rater reliability was not formally evaluated, which is a limitation of the study.

### Volume and thickness assessment

For the assessment of volume and thickness, the VolBrain [21] online segmentation tool was utilized. The Vol2Brain pipeline segmentation protocol, version 1.0 (accessed between 2024 and 2025), was employed for the quantitative analysis of the 3D T1-weighted MRI images. This pipeline facilitates automated analysis of brain MRI in both clinical and research settings, enabling multilayer anatomical segmentation of over 100 brain regions. It is based on fast, multi-scale, multi-atlas label fusion technology with systematic error correction, providing quantitative data. The platform delivers volumetric results as both absolute values and values normalized relative to intracranial volume (ICV). Consequently, the volumes and thicknesses of various brain regions, including individual gyri, are expressed in cubic millimeters (mm^3^) and cortical thickness in millimeters (mm). The final output includes a PDF report detailing the volume and thickness of the cortex for each gyrus and subcortical structure. Prior to uploading to the VolBrain platform, all MRI scans were anonymized, and segmentations were visually inspected for gross errors. The VolBrain platform automatically compares individual measurements against age- and sex-adjusted normative reference values derived from a healthy sample, which has been validated in large populations. Additionally, a small age-matched cognitively normal control group (*n* ═ 20) was included to assess the applicability of the normative cut-off thresholds and to establish the definition of minimal atrophy based on our scanner and acquisition protocol (Table S1).

Subsequent steps involved defining hippocampal and cortical regions of interest (ROIs) based on previously established regions [8]: the total hippocampal ROI and three cortical ROIs: the frontal ROI (total middle frontal gyri cortices), the temporal ROI (total superior temporal gyri cortices), and the parietal ROI (total angular and supramarginal gyri cortices). Since the Vol2Brain pipeline does not provide a direct inferior parietal lobule ROI, this region was operationalized using its two anatomical components (angular and supramarginal gyri); the parietal ROI was deemed atrophic when at least one of these structures exhibited abnormal values. An ROI was classified as atrophic if the measured volume or cortical thickness fell below the lower bound of the population-based normative range provided by the Vol2Brain pipeline. This system defines 95% normative reference limits adjusted for the subject’s sex and age, derived from an internal database of over 3,000 healthy controls. Operationally, the threshold for atrophy was established as any value below the 2.5th percentile of this distribution (Table S2). No additional post-processing thresholds or recalculations were implemented. Each ROI was subsequently classified as atrophic or non-atrophic.

To facilitate the comparison of volume- and thickness-based MRI phenotypes with scale-based methods, patients were grouped according to the pattern of affected structures. Typical phenotypes included hippocampal atrophy with concurrent atrophy in at least one cortical ROI. Hippocampal sparing was characterized by a preserved hippocampus alongside atrophy in any cortical ROI. The limbic predominant phenotype comprised isolated hippocampal atrophy without cortical involvement, while the minimal atrophy phenotype included patients without detectable atrophy in any examined ROI. The established rules were applied to define both volume- and thickness-based phenotypes, enabling comparisons across different phenotyping approaches. Classification was based on the presence or absence of atrophy in the examined ROIs according to predefined criteria; therefore, a separate category for borderline cases was not established.

### Ethical statement

The study was conducted in accordance with the Declaration of Helsinki and received approval from the Ethics Committee of the Medical Faculty, University of Niš, Serbia (decision no. 12-6422-2/3 dated 23 July 2020).

### Statistical analysis

Data are presented as standard descriptive statistics, including mean, standard deviation, and both absolute and relative frequencies. Comparisons of numerical variables among multiple phenotypes were conducted using analysis of variance (ANOVA) or the Kruskal–Wallis test, depending on data distribution. Associations between phenotypes were assessed using the chi-square test, while inter-method agreement was evaluated using Cohen’s kappa and Cramer’s V.

The primary analysis included comparisons of MRI phenotypes presented in Table 2, the association of different phenotypes with CDR and MMSE scores in Table 3, and comparisons of CDR components and MMSE groups across scale-based MRI phenotypes in Table 4 (only continuous data analysis). For all primary analyses involving multiple comparisons, *P*-values were adjusted using the Benjamini–Hochberg false discovery rate (FDR) procedure. The comparison of MMSE groups was retained as a supplementary clinical descriptor in Table 4 and was not part of the primary analysis. All other comparisons were regarded as exploratory analyses and were not subjected to FDR correction. The null hypothesis was tested with a significance threshold of *P* < 0.05. Statistical analyses were performed using the R software package version 4.5.1 [22].

**Table 2:** Agreement and association between scale-based and alternative MRI phenotyping methods

**Scale-based MRI phenotypes**		**Adjusted scale-based MRI phenotype**		**Volume-based MRI phenotype**		**Thickness-based MRI phenotype**	
		**Value**	***P***	**Value**	***P***	**Value**	***P***
Nominal by nominal	Cramer’s V	0.831	0.000	0.973	0.000	0.285	0.371
Measure of agreement	Kappa	0.429	0.000	0.957	0.000	0.150	0.164
	95% CI	0.204–0.654		0.874–1.000		--0.073–0.373	
Percent agreement		28/40	70%	39/40	97.5%	20/40	50.0%
*P* values for the Fisher’s exact test		<0.0001		<0.0001		0.383	
*P*.adjust (FDR)		<0.0001		<0.0001		0.479	

Adjusted *P* values (FDR) were calculated using the Benjamini–Hochberg procedure. Abbreviations: CI, confidence interval; FDR, false discovery rate; MRI, magnetic resonance imaging.

**Table 3:** Overlap of phenotypes with CDR and MMSE scores

**CDR vs.**		**Scale-based MRI phenotypes**		**Adjusted scale-based MRI phenotype**		**Volume-based MRI phenotypes**		**Thickness-based MRI phenotypes**	
		**Value**	***P***	**Value**	***P***	**Value**	***P***	**Value**	***P***
Nominal by nominal	Phi	0.473	0.030	--0.202	0.201	0.458	0.039	0.237	0.326
	Cramer’s V	0.473	0.030	0.202	0.201	0.458	0.039	0.237	0.326
Measure of agreement	Kappa	--0.095	0.020	--0.050	0.201	--0.095	0.020	--0.050	0.154
*P* values for Fisher’s exact test		0.016		0.281		0.022		0.378	
p.adjust (FDR)		0.080		0.421		0.083		0.479	
**MMSE vs.**		**Scale-based MRI phenotypes**		**Adjusted scale-based MRI phenotype**		**Volume-based MRI phenotypes**		**Thickness-based MRI phenotypes**	
		**Value**	***P***	**Value**	***P***	**Value**	***P***	**Value**	***P***
Nominal by Nominal	Phi	0.547	0.063	0.145	0.657	0.534	0.077	0.243	0.670
	Cramer’s V	0.387	0.063	0.145	0.657	0.378	0.077	0.172	0.670
Measure of Agreement	Kappa	--0.124	0.017	--0.046	0.385	--0.127	0.015	0.038	0.421
*P* values for the Fisher’s exact test		0.042		0.540		0.056		0.692	
p.adjust (FDR)		0.105		0.579		0.112		0.692	

Adjusted *P* values (FDR) were calculated using the Benjamini–Hochberg procedure. Abbreviations: CDR, Clinical Dementia Rating; FDR, false discovery rate; MMSE, Mini-Mental State Examination; MRI, magnetic resonance imaging.

**Table 4:** Comparison of CDR measures, MMSE scores, and group assignments among scale-based MRI phenotypes

**Characteristics**	**Typical**		**Limbic predominant**		**Hippocampal sparing**		**Minimal atrophy**		***P***	**p.adjust (FDR)**	**Effect size**
	***n* ═ 7**		***n* ═ 2**		***n* ═ 23**		***n* ═ 8**	
CDR-SOB	13.07±2.09		9.00±8.49		9.09±3.63		7.56±4.82		0.061^2^	0.112	0.121^3^
											Medium
CDR global score	2.14±0.38		1.75±1.77		1.48±0.65		1.19±0.88		0.067^2^	0.112	0.116^3^
											Medium
MMSE score	17.00±5.03		14.50±12.02		19.78±5.01		20.13±6.48		0.488	0.563	0.000^3^
											Negligible
MMSE groups											
I	2	28.6	1	50.0	12	52.2	6	75.0			0.387^4^
II	4	57.1	0	0.0	11	47.8	1	12.5	0.042^1^	0.105	Moderate
III	1	14.3	1	50.0	0	0.0	1	12.5			

MMSE groups: I: mild, II: moderate, III: severe; ^1^ Fisher’s exact test; ^2^ Kruskal–Wallis test; ^3^ effect size was quantified using η^2^ based on the Kruskal–Wallis H statistic; ^4^ Cramer’s V. FDR-adjusted *P* values were calculated using the Benjamini–Hochberg procedure. Abbreviations: CDR, Clinical Dementia Rating; CDR-SOB, Clinical Dementia Rating–Sum of Boxes; FDR, false discovery rate; MMSE, Mini-Mental State Examination; MRI, magnetic resonance imaging.

## Results

This study comprised 40 patients (18 male and 22 female), with an average age of 68.88 ± 7.75 years (range 53–81 years). Across all methods for determining MRI phenotypes, the most prevalent phenotype in our cohort was the hippocampal sparing phenotype, with prevalence rates ranging from 50% (adjusted scale-based method) to 75% (thickness-based MRI method). Demographic and clinical characteristics did not significantly differ across MRI phenotypes defined by scale-based and volume-based methods (Tables S3 and S4).

Comparison of MRI phenotyping methods relative to the scale-based approach revealed a strong, statistically significant association between scale-based and adjusted scale-based MRI phenotypes (Cramer’s *V* ═ 0.831, *P* < 0.001), with moderate agreement between categories (Kappa = 0.429). A statistically significant association was also observed between scale-based MRI phenotypes and volume-based MRI phenotypes (Cramer’s *V* ═ 0.973, *P* < 0.001), demonstrating excellent agreement (Kappa = 0.957) (Table 2). In contrast, no statistically significant association or agreement was observed between scale-based and thickness-based MRI phenotypes (Cramer’s *V* ═ 0.285, *P* ═ 0.371; κ ═ 0.150, *P* ═ 0.164) (Table 2). Percent agreement was 70% for scale-based and adjusted scale-based MRI phenotypes, 97.5% for scale-based and volume-based phenotypes, and 50.0% for scale-based and thickness-based phenotypes. Although Cohen’s kappa indicated moderate agreement among observed phenotypes, the wide 95% confidence interval, percent agreement, and cross-tabulations (Table S5) suggest that this agreement is primarily influenced by the predominance of the hippocampal sparing phenotype.

We analyzed the overlap of phenotypes across four phenotyping methods, including CDR and MMSE scores, as well as clinical phenotypes. The CDR score was found to be statistically significantly associated with scale-based MRI phenotypes (*P* ═ 0.016) and volume-based MRI phenotypes (*P* ═ 0.022); however, neither association remained significant after FDR correction (*P* ═ 0.080, *P* ═ 0.083, respectively). The strength of association in these comparisons was near the upper limit of moderate association (Cramer’s *V* ═ 0.473 and 0.458). Although the Cohen’s kappa value was statistically significant, its findings are limited due to the asymmetry of the comparison. No statistically significant association was observed between MMSE scores and MRI phenotypes following correction for multiple comparisons (Table 3).

The distribution of CDR-SOB and CDR Global Score did not differ significantly across MRI phenotypes (*P* ═ 0.061 and *P* ═ 0.067), although medium effect sizes were noted (η^2^ ═ 0.121 and η^2^ ═ 0.116, respectively) (Table 4). Total MMSE scores also did not differ significantly across the examined phenotypes (*P* ═ 0.488), with a negligible effect size (η^2^ ═ 0.000). Although the distribution of MMSE groups varied across MRI phenotypes (*P* ═ 0.042), indicating a moderate ordinal association (Cramer’s *V* ═ 0.387), this association did not retain statistical significance after FDR correction (*P*.adjust = 0.105).

The majority of patients exhibited an amnestic phenotype (*n* ═ 24, 60.0%), while non-amnestic presentations were observed in 16 cases (40.0%). Non-amnestic presentations included frontal variant Alzheimer’s disease (*n* ═ 3, 7.5%), posterior cortical atrophy (*n* ═ 5, 12.5%), and logopenic variant primary progressive aphasia (*n* ═ 8, 20.0%). Clinical phenotypes were not significantly associated with the examined MRI phenotypes (χ^2^ tests, *P* ═ 0.288–0.470), and no meaningful overlap in findings was observed.

To verify the normative thresholds provided by the automated pipeline on our scanner and acquisition protocol, we also analyzed a small age-matched cognitively normal sample (*n* ═ 20). As anticipated, the majority of controls were classified as minimal atrophy by both volume-based and thickness-based phenotyping approaches (19/20 and 14/20, respectively), whereas minimal atrophy was less frequent in the AD cohort (7/40 and 12/40, respectively) (Table S1).

## Discussion

A comparison of MRI phenotyping methods relative to the scale-based approach indicated a statistically significant agreement between the scale-based and adjusted scale-based MRI phenotypes in this sample. A statistically significant association was also observed between scale-based and volume-based MRI phenotypes. The only notable difference in agreement was found for the thickness-based method compared to the other approaches. This discrepancy may be attributed to the higher proportion of the hippocampal sparing phenotype identified by the thickness-based method (up to 75%), which alters the distribution of categories and reduces concordance with other methods. Our findings can be partially elucidated by the work of Clerx et al. [23], who demonstrated that cortical thickness is more sensitive than volumetric methods in the early stages of AD. Similar results were reported by Matsuda [24], who emphasized that standard volumetric analyses of subcortical structures, such as the hippocampus, yield more reliable quantitative data. In the context of our findings, the heightened sensitivity of cortical thickness measurements may facilitate the more frequent identification of the hippocampal sparing phenotype, as cortical changes can be detected prior to significant hippocampal atrophy. Our results reveal variable agreement between MRI phenotyping approaches, with moderate agreement observed for adjusted scale-based methods and excellent agreement for volume-based methods. Consistent with this, Ferreira et al. [20] demonstrated that adjusted cut-offs and adjusted scale-based MRI phenotypes may enhance diagnostic and prognostic performance, as well as patient selection for clinical trials. However, these approaches necessitate additional information, such as APOE genotype and age at disease onset, which may not be readily available or may be obtained later, thereby limiting their practical application. Collectively, these findings advocate for the use of simplified visual rating approaches in clinical settings. Given that volumetric analysis is not widely accessible and requires additional resources, while visual scales based on standard cut-off values do not necessitate APOE genotype or information on disease onset, MRI phenotyping may be feasibly integrated into routine clinical practice using simple visual rating scales.

The dominant prevalence of the hippocampal sparing phenotype is consistently observed across various phenotyping methods. The existing literature indicates that the most common MRI phenotypes are typical, followed by limbic predominant, hippocampal sparing, and minimal atrophy [7, 25]. However, in our sample, we noted a consistently higher frequency of the hippocampal sparing phenotype across all phenotyping methods, ranging from 50% in the scale-based approach to 75% in the thickness-based approach. Aligning with our findings, Persson et al. [7] reported that the hippocampus was not atrophied in 60% of cases; however, that study did not examine the four classic MRI phenotypes. The patients in their study were younger and exhibited less global cognitive impairment, particularly in memory and abstract functions, but demonstrated weaker performance in executive, visuospatial, and semantic tasks, along with a higher prevalence of the non-amnestic form of AD compared to those with hippocampal atrophy. Additionally, the potential influence of sample selection and the demographic characteristics of our study area on the relative frequency of the hippocampal sparing subtype of AD cannot be discounted. Certainly, further longitudinal studies are warranted to elucidate the factors influencing the prevalence of hippocampal sparing and its clinical implications.

In our investigation, characteristics such as sex, age, education, APOE status, and time since symptom onset did not significantly differ between phenotypes defined by scales and volume. Similarly, Wheatley et al. [26] did not identify a statistically significant relationship. In contrast, longitudinal studies conducted by Machado et al. [27] and Inguanzo et al. [28] indicated that demographic and genetic factors may significantly associate with MRI phenotypes of AD. Additionally, Persson et al. [7] reported that patients with minimal atrophy had fewer years of formal education, supporting the cognitive reserve hypothesis.

Although the distribution of MMSE scores initially appeared to differ across MRI phenotypes, this association did not retain statistical significance after correction for multiple comparisons. Consequently, our findings do not provide robust evidence for a correlation between cognitive severity (measured by MMSE) and MRI phenotype classification. This contrasts with the findings of Byun et al. [8], who reported statistically significant differences in cognitive status, with the highest MMSE scores observed in subjects with minimal atrophy.

Regarding CDR values, we found no statistically significant differences between phenotypes determined by scales in our sample. However, a pattern emerged wherein the typical and limbic predominant phenotypes tended to have higher (worse) CDR scores, while minimal atrophy and hippocampal sparing demonstrated better performance, suggesting a potential trend that did not reach statistical significance. The relatively preserved function in the hippocampal sparing phenotype may reflect an earlier stage or a specific point in the disease progression, rather than serving as a direct diagnostic marker. Persson et al. [7] observed significant differences between phenotypes, but, unlike our findings, better performance was noted in patients who did not develop hippocampal atrophy.

A limitation of MRI phenotyping is that phenotypes are determined without explicit consideration of disease stage. The emerging Subtype and Stage Inference (SuStaIn) method [29] is a machine-learning technique that combines MRI phenotype with the temporal progression pattern, thereby simultaneously incorporating phenotyping and staging of subjects. SuStaIn assigns each MRI subtype or phenotype a probable disease stage, potentially explaining why some clinical tests correlate more strongly with certain phenotypes [29, 30].

### Limitations

This study has several limitations that warrant consideration. First, the relatively small sample size may restrict the generalizability of the results and diminish statistical power, particularly in subgroups with rare MRI phenotypes. Another limitation is that visual rating scales were assessed by a single experienced neuroradiologist; hence, inter-rater reliability was not evaluated. We defined MRI phenotypes using automated volumetric and cortical thickness measurements. Although these methods have been validated in prior research, they may still exhibit variability in how they segment brain structures. Furthermore, a formal sensitivity analysis was not conducted, as the study’s objective was not to evaluate alternative thresholds. Subsequent analyses should explore whether alternative thresholds may influence phenotype classification. Nonetheless, the inclusion of a small age-matched cognitively normal control group confirmed that the normative thresholds behaved as expected on our scanner and protocol. However, this sample was not intended to represent a fully characterized control cohort, as laboratory assessments and detailed neuropsychological testing were not systematically performed in these individuals. Additionally, the partial-volume effect may limit MRI-based morphometric measurements, particularly in the analysis of smaller brain structures. Finally, because our study captured data at a single point in time, we cannot draw conclusions about long-term disease progression or whether these phenotypes remain stable as the condition evolves.

## Conclusion

Our findings suggest that scale-based MRI phenotyping exhibits moderate agreement with adjusted scale-based approaches and high agreement with volumetric methods in this Serbian cohort. These results indicate that visual scale-based phenotyping may represent a practical approach to MRI phenotyping in resource-limited clinical settings where advanced methods are unavailable. However, these findings should be interpreted with caution and require validation in larger independent cohorts. Further research is necessary to examine the relationship between thickness- and scale-based phenotyping methods, particularly across different disease stages.

The lack of significant agreement with demographic variables and the differences observed across CDR domains highlight the clinical heterogeneity of phenotypes. Discrepancies in our results concerning demographics, APOE status, and clinical phenotypes in this Serbian sample, in comparison with findings reported in other populations, warrant further investigation.

## Supplemental data

Supplemental data are available at the following link: https://www.bjbms.org/ojs/index.php/bjbms/article/view/13974/4162.

## Data Availability

Data are available from the corresponding author upon reasonable request.
